# SAFESLOT: an experimental virtual reality protocol to examine flow, craving, and cognitive biases in gambling

**DOI:** 10.3389/fpsyt.2026.1787985

**Published:** 2026-05-08

**Authors:** Azzurra di Palma, Miranda Bellezza, Sofia Santisi, Rosaria Giordano, Andrea Frosini, Elisa Pergola, Caterina Primi, Maria Anna Donati

**Affiliations:** 1 Department of Mathematics and Informatics, “Ulisse Dini” (DiMAI), University of Florence, Florence, Italy; 2 Department of Statistics, Computer Science, Applications “Giuseppe Parenti” (DiSIA), University of Florence, Florence, Italy; 3 Department of Neuroscience, Psychology, Drug, and Child’s Health (NEUROFARBA), University of Florence, Florence, Italy

**Keywords:** biofeedback, cognitive distortions, craving, gambling disorder, machine learning, research protocol, slot machine, VR environment

## Abstract

Gambling-related harm comes out from dynamic interactions between craving, cognitive distortions, and physiological responses to gambling outcomes. These processes happen rapidly during play and are strongly influenced by the structural features of slot machine, yet they are seldom captured in real time using ecologically valid methods. Traditional research has relied mainly on self-report measures and simplified gambling simulations, limiting the ability to assess moment-to-moment changes in motivation, cognition, and arousal. The present study aims to address these limitations by developing SAFESLOT: a virtual slot machine designed *ad hoc* to examine behavioral and psychophysiological mechanisms underlying gambling disorder risk and to test whether interrupting gambling activity can attenuate craving and cognitive distortions. The study will recruit approximately 200 university students who will participate in a controlled virtual reality slot-machine task. The virtual environment replicates a realistic Video Lottery Terminal. The task will be composed by five gameplay sessions, each emphasizing a distinct feature of slot machines. Participants will be randomly assigned to one of three conditions: uninterrupted gambling, gambling interrupted by brief reflective pauses with questions concerning craving and perceived winning probability, and gambling interrupted without self-reflective prompts. During the task, continuous multimodal data will be collected, including psychophysiological signals, behavioral indicators such as reaction times, betting patterns, and eye-tracking measures. Psychological self-report assessments will be administered before and after the virtual gambling experience. Data analysis will combine classical statistical methods with machine learning techniques in order to identify patterns associated with changes in craving and gambling-related cognitive distortions. Our study aims to clarify how specific gambling outcomes influence craving, cognition, and arousal. The comparison between uninterrupted and interrupted gambling conditions will offer insights into the potential preventive value of brief reflective pauses in reducing automatic gambling responses. The findings may contribute to the development of evidence-based prevention and responsible gambling interventions.

## 1 Background

Gambling has become a pervasive component of contemporary entertainment culture, supported by widespread accessibility to both physical venues and online platforms (1). Although often framed as a leisure activity, gambling poses substantial public health concerns due to its potential to develop into Gambling Disorder (GD), a psychiatric condition characterized by persistent, maladaptive gambling behaviors associated with marked distress and functional impairment (2, 3). Understanding the mechanisms that support gambling behavior remains a priority for prevention and early diagnosis.

Gambling-related harm arises from the interplay between individual vulnerabilities, the structural characteristics of gambling products, and environmental accessibility (4–6). Craving and cognitive distortions represent key drivers of sustained gambling behavior (7, 8). Craving is defined as an intrusive and urgent desire to continue gambling (3), engaging mesolimbic circuits including the ventral striatum and other reward-sensitive regions (9, 10). These processes are rapid, dynamic, and tightly contingent on individual game outcomes, making them difficult to capture with traditional trait-focused self-report measures. The instantaneous nature of these neurocognitive responses justifies the use of continuous psychophysiological measures to capture moment-to-moment variations in arousal, motivation, and attentional biases.

Electronic Gaming Machines (EGMs), particularly slot machines, incorporate structural features known to enhance engagement, cognitive distortions, and physiological arousal. Near misses, large initial wins, large losses, small losses, and frequent small wins produce intermittent reward patterns that disproportionately reinforce gambling behavior (1, 11–13). Neurophysiological evidence shows that near misses activate the ventral striatum almost as strongly as wins (9) and elicit autonomic responses exceeding those associated with either wins or losses (12). Similarly, large wins and Losses Disguised as Wins (LDWs) distort probability perception and reduce awareness of net losses (14).

However, current research investigating cognitive distortions and craving during gambling remains limited by several methodological constraints. First, most studies rely on self-report instruments such as the Gambling Related Cognitions Scale (GRCS) (8) and Visual Analogue Scales (VAS) (15) for craving, which primarily capture trait-level tendencies and lack sensitivity to state-dependent fluctuations that arise during actual gambling episodes (7, 16, 17). Second, experimental paradigms often use simplified or non-immersive gambling simulations, such as two-dimensional slot machines displayed on standard monitors (14, 18–22), which constrain ecological validity and fail to reproduce the sensory and attentional conditions characteristic of real-world gambling environments (11). Third, while some recent work has incorporated Virtual Reality (VR), the only study that simulated slot machines in VR (11) examined solely near-miss effects on heart rate and post-reinforcement pause, without considering other structural features or multimodal assessment. Additionally, studies combining VR with physiological measures (23–25) suffer from small samples and inadequate assessment of presence and immersion (26). To our knowledge, no previous study has combined immersive VR, continuous multimodal measurement including physiological monitoring, behavioral tracking, and eye-tracking, together with systematic manipulation of specific slot machines structural features within a single protocol. This gap is particularly pronounced for university students, an accessible yet understudied population showing increasing rates of online gambling engagement (27).

The present study is developed to bridge this gap through SAFESLOT (Safe Assessment Framework for Electronic SLot-machine Online Testing), a multimodal assessment protocol designed to monitor behavioral, psychological, and physiological responses during simulated gambling. The protocol uses a custom-designed VR platform that replicates slot machine gameplay within a controlled but ecologically valid 3D environment. Five distinct gambling sessions emphasize distinct slot machine structural features: near misses, large initial wins, large losses, small losses, and frequent small wins, allowing systematic examination of their differential impact on cognition and affect.

The protocol integrates three primary data streams:

- Psychophysiological monitoring (skin conductance, photoplethysmography, eye-tracking);
- Behavioral metrics (betting patterns, decision latencies);
- Psychological assessments (impulsivity, gambling-related cognitions, GD severity, distress symptoms) (28, 29).

Participants will be randomly assigned either to a Flow Group, experiencing uninterrupted gambling, to a Reflection Group, in which gambling is periodically interrupted by self-reflective prompts assessing craving and perceived winning probability, or to a Break Group that will follow the same procedure as the Reflection Group, but a loading screen will appear between sessions instead of the self-reflective prompts. This design will allow us to disentangle the effects of measurement from the effects of taking breaks, ensuring that any observed differences between the Flow Group and the Reflection Group can be attributed specifically to the presence of self-reflective prompts rather than to the interruptions themselves. This approach enables identification of physiological and behavioral markers associated with changes in cognitive distortions and craving from pre- to post-experience, specifically distinct values or ranges of skin conductance response amplitude, heart rate variability, pupil diameter, and fixation duration. Such markers would provide objective indicators of gambling-related risk detectable in real time and test whether brief reflective pauses can reduce cognitive distortions and craving, offering a potential preventive strategy.

The present study aims to identify specific physiological and behavioral patterns that correspond to increases or decreases in gambling-related cognitive distortions and craving from pre- to post-exposure, and to test whether interrupting gambling with self-reflective prompts attenuates these changes compared to continuous play. To this end, self-report measures will be collected before and after the gambling sessions, while physiological, oculometric, and behavioral indices will be continuously recorded during play.

To enhance conceptual clarity, we distinguish between primary confirmatory hypotheses and secondary exploratory analyses. Primary hypotheses focus on the effects of gambling interruption (Flow vs. Reflection vs. Break conditions) on craving, cognitive distortions, and behavioral reactivity. Specifically, we expect that: the Flow Group will exhibit enhanced craving and cognitive distortions compared to the Reflection Group at post-test, the Break Group will show intermediate effects and interruptions will attenuate automatic psychophysiological and behavioral responses to gambling outcome. According to these confirmatory aims, we further hypothesize that physiological activation, indexed by skin conductance response amplitude and reduced heart rate variability, will negatively correlate with reaction times following gambling outcomes, with associations varying by outcome type. Specifically, near misses and large wins are expected to produce prolonged reaction times despite high arousal due to attentional capture, whereas losses are expected to show stronger negative associations reflecting rapid escape responses. We further predict that these arousal-behavior coupling patterns will be more pronounced in the Flow Group compared to the Reflection Group, as interruptions should attenuate automatic psychophysiological reactivity. We expect the Break Group to show intermediate patterns, with arousal/behavior coupling less attenuated than in the Reflection Group but not as pronounced as in the Flow Group, reflecting the effect of interruptions without measurement. Additionally, we hypothesize that participants showing increases in craving and cognitive distortions from pre-test to post-test will exhibit higher physiological arousal and shorter reaction times during play, constituting a high-risk psychophysiological profile. Finally, we predict that the Flow Group will exhibit enhanced craving compared to the Reflection Group at post-intervention assessment, with a between-group difference of at least Cohen’s d = 0.5 on craving reduction, controlling for baseline equivalence in gambling behavior, cognitive distortions, craving, impulsivity, and distress symptomatology. We further expect the Break Group to show intermediate effects, with craving reduction, greater than in the Flow Group, but less pronounced than in the Reflection Group. In parallel, secondary analyses are exploratory and aim to characterize complex multimodal patterns. In particular, eye-tracking measures (gaze direction, fixation duration, pupil diameter) will be examined to identify attentional and autonomic correlates of gambling outcomes and their relationship with craving and cognitive distortions. Additionally, machine learning approaches (unsupervised clustering and supervised neural networks) will be used to identify latent response profiles and explore the predictive value of integrated behavioral and physiological data. These analyses are intended to generate hypotheses for future research rather than to test predefined directional predictions. Individual differences in impulsivity and psychological distress (depression, anxiety, and stress symptoms) will be assessed and statistically controlled, given their established association with gambling-related problems. To characterize multimodal response patterns, the study combines classical statistical approaches including correlational analyses and mixed ANOVAs with machine learning methods to identify latent subgroups and to predict gambling-related risk profiles from physiological and behavioral data.

## 2 Methods

### 2.1 Virtual environment

**Figure 1:**
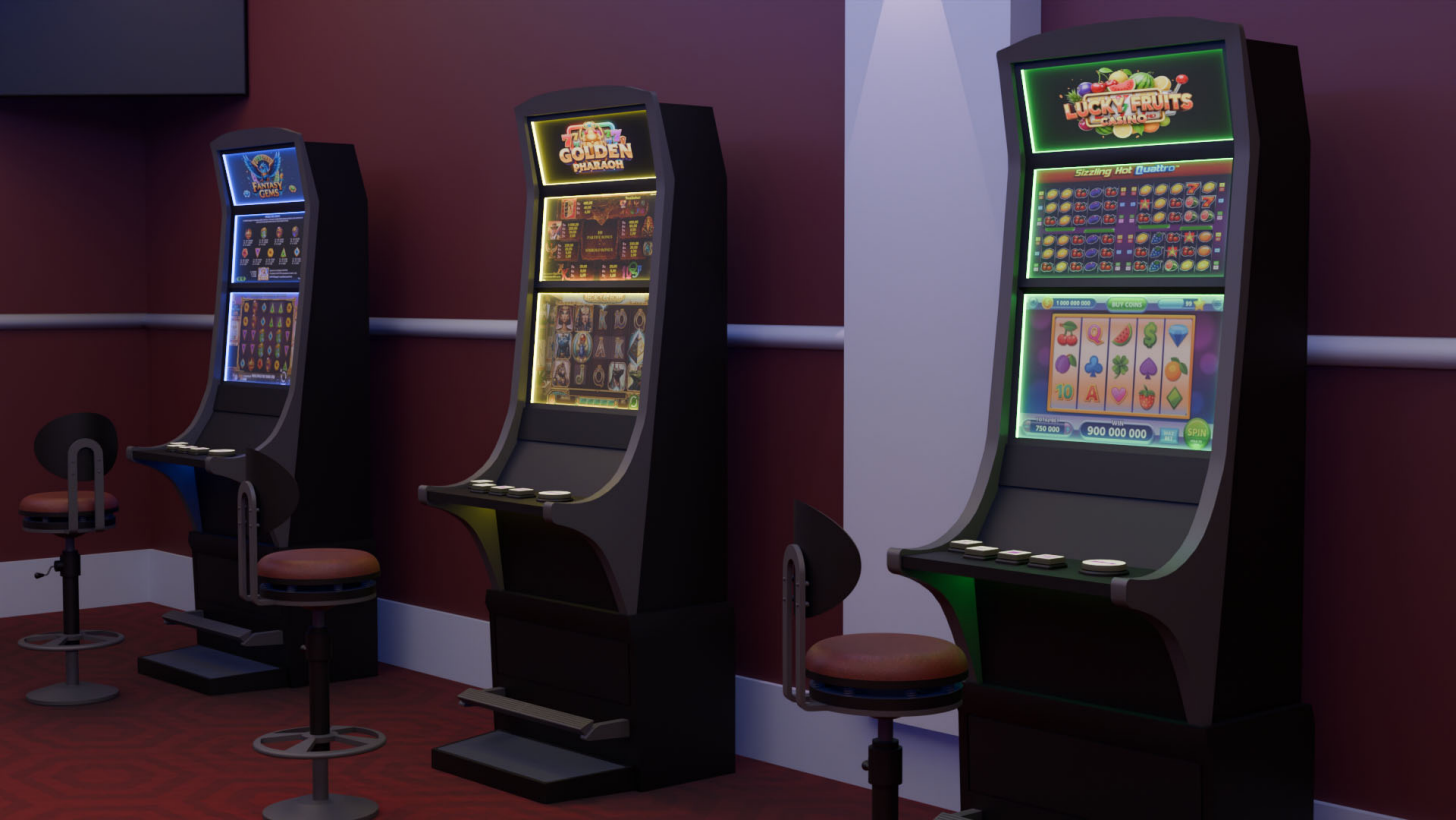
West side of the SAFESLOT slot machine room. The figure shows the west-facing wall and part of the experimental environment used in the VR simulation.

The SAFESLOT protocol employs a custom-designed immersive virtual reality (VR) environment that simulates a realistic video lottery terminal (VLT) setting (see **Figure 1**). Participants interact with a virtual slot machine while seated, ensuring standardized posture and minimizing cybersickness (30).

The SAFESLOT system will integrate VR hardware with multimodal physiological recording. Visual immersion and gaze behavior will be captured using the Vive Focus Vision Headset equipped with integrated eye tracking. Autonomic nervous system activity will be continuously monitored using the BITalino (r)evolution Biosignals Platform with photoplethysmography (PPG) and electrodermal activity (EDA) sensors.

Additional technical specifications, hardware details, and visual materials are provided in Appendix.

### 2.2 Software description

**Figure 2:**
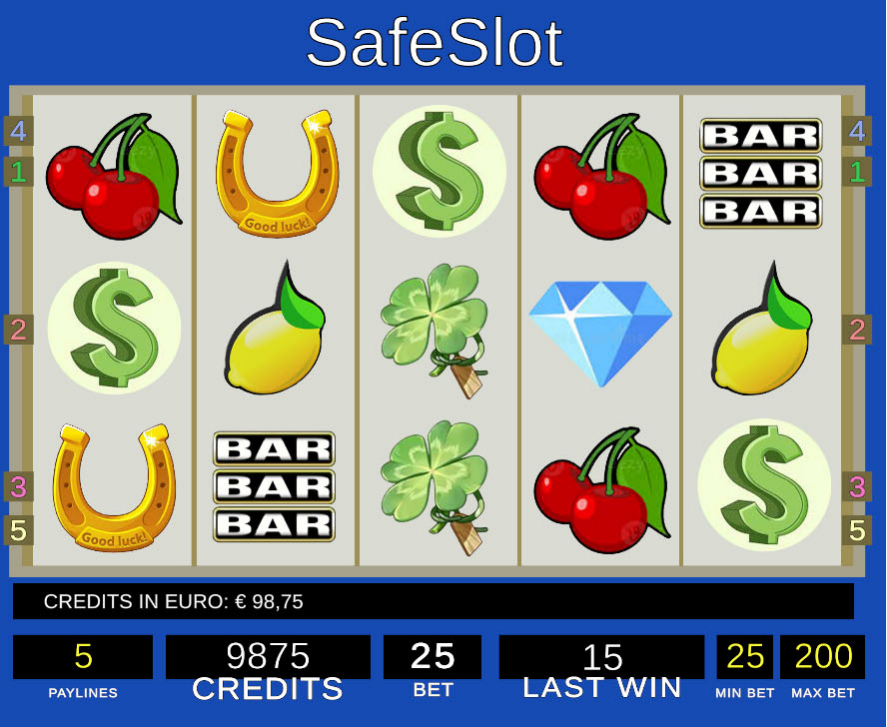
SAFESLOT interface showing the main screen.

**Figure 3:**
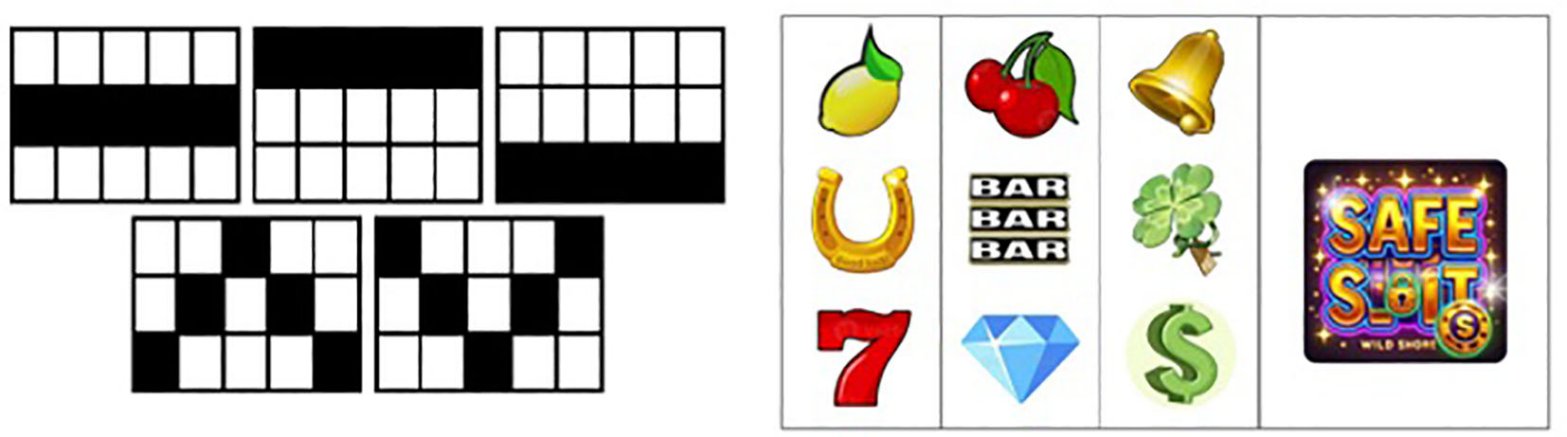
On the left: Schematic view of active paylines in the SAFESLOT virtual slot machine. Each line represents a different winning pattern across the 3*×*5 reel matrix. On the right: The nine standard symbols and thew wild symbol (on the right) used in the SAFESLOT machine.

SAFESLOT software simulates a VLT room with some slot machines where only one is active that adheres to the classic configuration of five reels with three consecutive visible symbols each, standard control panel, and five paylines as represented in **Figures 2**, **3**, left-side. The symbols are also standard, as depicted in **Figure 3**, right-side. A special wild symbol is also presented, whose role is both generating the super jackpot and substituting any other symbol to complete a winning configuration (see more details in Appendix).

Participants interact with the machine through VR-based hand tracking. This allows them to adjust bets, active paylines, access general info such as payout tables and game rules, and, finally, initiate spins.

Each spin has a fixed duration of 3 seconds, to ensure a controlled experimental setting across all participants with the aim of isolating and examining the variables of interest with greater internal validity.

Winning symbol configurations and associated payouts in the SAFESLOT machine are depicted in **Table 1**.

**Table 1:** The figure displays all adopted winning configurations.

Symbols	Aligned	Credits			
		25	50	100	200
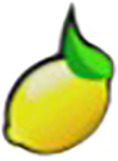 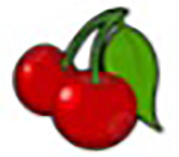	x3	15	30	60	120
	x4	30	60	120	240
	x5	45	90	180	360
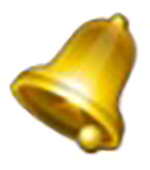 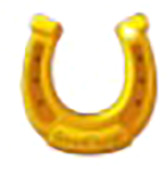	x3	25	50	100	200
	x4	50	100	200	400
	x5	75	150	300	600
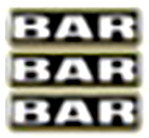 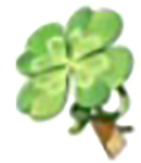	x3	50	100	200	400
	x4	100	200	400	800
	x5	150	300	600	1200
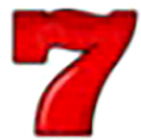	x3	100	200	400	800
	x4	200	400	800	1600
	x5	300	600	1200	2400
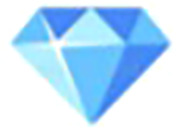	x3	150	300	600	1200
	x4	300	600	1200	2400
	x5	450	900	1800	3600
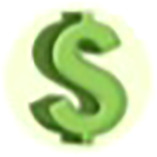	x3	200	400	800	1600
	x4	400	800	1600	3200
	x5	600	1200	2400	4800
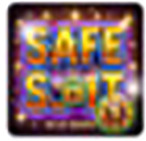	x5	1250	2500	5000	10000

The *Aligned* column indicates the number of identical symbols aligned along an active payline. The *Credits* columns report, from left to right, the payout values corresponding to bets of 25, 50, 100, and 200 credits, respectively.

Researchers can adjust spin sequences and outcome distributions, including psychologically relevant events such as near-misses, as well as customize the overall session length and incorporate post-session questionnaires that appear directly within the virtual environment. This functionality enables the precise manipulation of gambling-related stimuli and the consistent collection of data between participants. SAFESLOT consists of five game sessions, each consisting of 30 spins, and an initial tutorial session allowing participants to familiarize themselves with how the slot machine works through a few trial spins. Each gaming session is designed to emphasize a specific structural feature of the slot machine (**Table 2**): Near misses, large wins, losses, frequent LDWs, frequent small wins.

**Table 2:** Spin outcome distribution across five experimental SAFESLOT sessions.

Session type	Near misses	LDWs	Losses	Small wins	Big wins
Near Misses	32.6%(10)	30%(9)	26.7%(8)	10.7%(3)	0%(0)
Initial Big Wins	16.7%(5)	30%(9)	33.3%(10)	10%(3)	10%(3)
Frequent Losses	16.7%(5)	30%(9)	33.3%(10)	20%(6)	0%(0)
Frequent LDWs	16.7%(5)	50%(15)	10%(3)	20%(6)	3.3%(1)
Frequent Small Wins	16.7%(5)	16.7%(5)	6.7%(2)	56.6%(17)	3.3%(1)

Percentages and absolute frequencies are reported.

### 2.3 Virtual slot machine display elements

The virtual slot machine interface replicates the layout and functionality of commercial slot machines.

All display elements employed are described in **Table 3**. They are designed to clearly understand typography and colors and ensure readability within the virtual environment. The icons with information are the same as in commercial slot machines; this guarantees ecological validity and ensures that participants can monitor their gambling activity in real-time. Moreover, all interface interactions are logged with millisecond precision, capturing button press timing, bet selection patterns, use of max bet features, and response latencies to different gambling outcomes, providing comprehensive behavioral data for analysis of gambling behavior patterns and decision-making processes.

**Table 3:** Description of the virtual slot machine display elements.

Elements	Description
*Credits (€)*	Displays the participant’s current balance expressed in euros. This monetary representation provides immediate feedback on net gains or losses throughout the session, making financial consequences salient despite the use of virtual currency.
*Credits (Numerical)*	Shows the raw credit count available for wagering. This display updates in real time after each spin, increasing following wins and decreasing after losses, thereby providing continuous feedback on gambling outcomes independent of monetary value.
*Paylines*	Indicates the number of active paylines (**Figure 3**), representing the symbol patterns across the reel matrix along which winning combinations are evaluated.
*Bet*	Displays the current wager amount, expressed in credits, for the upcoming spin. The prominence of this element ensures participants remain aware of their risk level on each trial.
*Last Win*	Shows the credit amount won on the most recent winning spin. If the current spin results in a loss, this value is not reset to zero but continues to display the previous win.
*Min Bet*	Indicates the minimum allowable wager, providing a reference point for conservative betting strategies and reinforcing awareness of the available betting range.
*Max Bet*	Displays the maximum allowable wager, highlighting the upper limit of risk-taking behavior. The visual salience of this information may influence betting decisions, particularly following near misses or winning outcomes.

**Figure 4:**
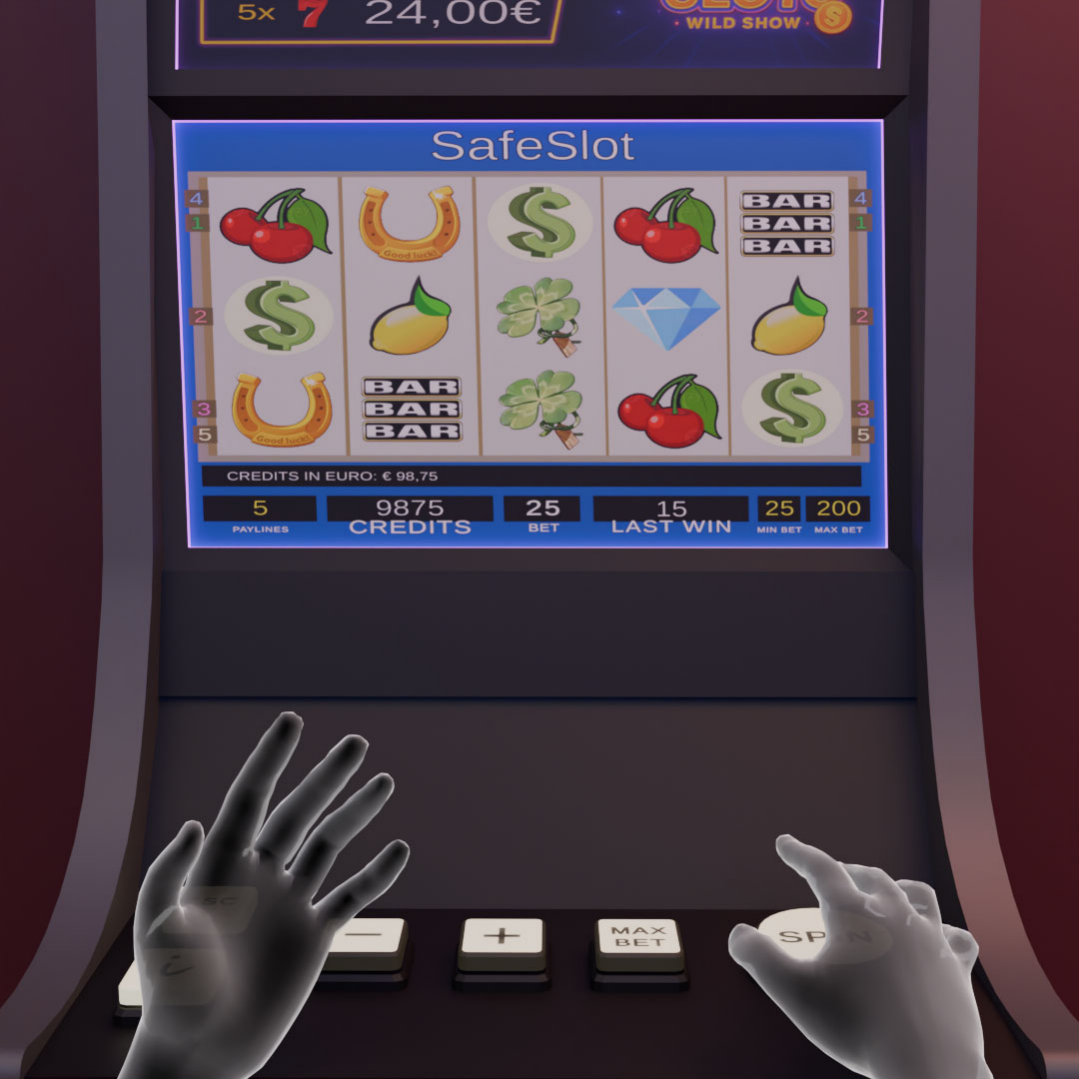
SAFESLOT machine interface. The image shows the perspective from player point of view.

The layout and updating logic of all these display elements are standardized across participants and sessions, ensuring that observed differences in gambling behavior reflect individual and experimental factors rather than interface variability. In the figure below is shown SAFESLOT machine from the player point of view (**Figure 4**).

### 2.4 Study design

**Figure 5:**
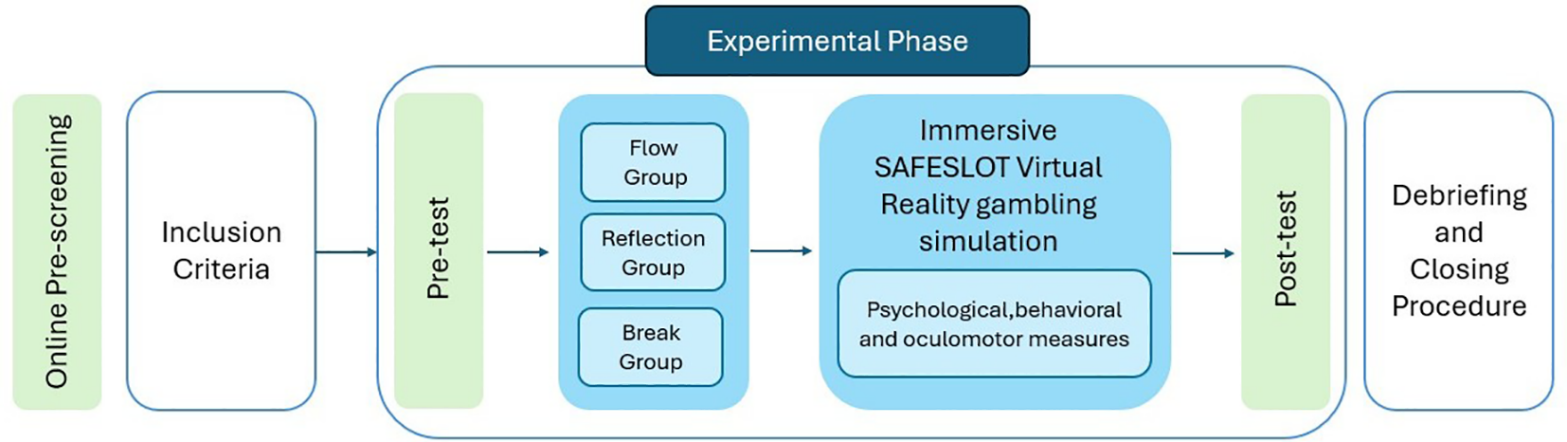
Overview of study design and experimental procedures.

This study will employ a multimodal design to investigate the effects of immersive virtual reality gambling exposure on behavioral, physiological, and cognitive responses in university students. The research protocol will consist of sequential phases as illustrated in **Figure 5**.

#### 2.4.1 Online pre-screening and inclusion criteria

All potential participants will complete an online screening battery (reported in **Table 4**) assessing socio-demographic characteristics, medical contraindications for VR exposure, and indicators of problematic or at-risk gambling. The protocol will be administered through a secure and anonymized platform in compliance with GDPR (General Data Protection Regulation) requirements and institutional ethical standards. Potential participants will access the study using a dedicated link distributed through official University of Florence channels. Participants who will not meet inclusion criteria are excluded from the experimental session but will receive the psycho-educational video and debriefing to ensure provision of preventive content to all screened individuals. Eligible individuals will be granted access to a digital scheduling system, allowing them to select their preferred laboratory appointment for a session lasting approximately 60–70 minutes.

**Table 4:** Overview of measures included in the online pre-screening phase.

Measure	Description
Socio-demographic Questionnaire	Age, gender, education level, academic program, civil and employment status, relevant background information. Standard categorical and open-response items.
Medical History	Systematic assessment of VR-related risk factors: epilepsy, hearing/vision impairments, cardiovascular conditions, vestibular dysfunction, migraines, neurological conditions. Binary and multi-option items.
Canadian Problem Gambling Index (CPGI) (31, 32)	9 items, 4-point frequency scale (0 = Never to 3 = Almost always). Screening for gambling-related risk.
SCL-90 Psychoticism Subscale (33, 34)	10 items, 5-point intensity scale (0 = Not at all to 4 = Extremely). Measures social withdrawal, isolation, and psychoticism.
Family Gambling History	Binary assessment (Yes/No) of gambling problems in family members, followed by forced-choice items identifying gambling activities.
Technology Familiarity Assessment	5 items adapted from the ITC-SOPI: VR exposure (Yes/No), stereoscopic 3D experience (Yes/No), computer proficiency (0–3), VR knowledge (0–3), gaming frequency (0–3).
Ten-Item Personality Inventory Revised (TIPI-R) (35, 36)	10 items, 7-point agreement scale (1 = Strongly disagree to 7 = Strongly agree). Measures Extraversion, Agreeableness, Conscientiousness, Emotional Stability, and Openness.
Sixteen-Item Balanced Inventory of Desirable Responding (BIDR) - 6 short version (37, 38)	16 items, 6-point Likert-type response scale (1 = Strongly disagree to 6 = Strongly agree). Measures Self-Deceptive Enhancement and Impression Management.

#### 2.4.2 Experimental phase

Upon arrival, participants will provide written informed consent and receive a standardized briefing on possible risks, including cybersickness and emotional discomfort. Then, they will complete a comprehensive pre-test battery including trait-level psychological constructs (e.g., impulsivity, distress) and state-dependent measures relevant to gambling motivation and cognition. The related questionnaires are reported in **Table 5**. A state measure refers to a temporary emotional or psychological condition that can fluctuate depending on situational factors, whereas a trait measure captures stable and enduring individual characteristics. Participants are allocated to one of three experimental conditions (Flow, Reflection or Break Group) using a computer-generated random seed with stratification to balance key individual-difference variables.

**Table 5:** Trait and state psychological measures administered during pre-test and post-test assessments.

Measure	Description
Trait measures	
Depression Anxiety Stress Scales-21 (DASS-21) (39, 40)	21-item measure of psychological distress rated on a 4-point frequency scale (0 = “Did not apply to me at all” to 3 = “Applied to me very much, or most of the time”). Includes three subscales: Depression, Anxiety, and Stress.
Short UPPS-P Impulsive Behavior Scale (41, 42)	20-item multidimensional assessment of impulsivity using a 4-point agreement scale. Subscales include Positive Urgency, Negative Urgency, Lack of Premeditation, Lack of Perseverance, and Sensation Seeking.
GRCS–Revised for Adolescents (GRCS-RA) (8, 43)	14-item measure assessing gambling-related cognitive beliefs on a 5-point agreement scale, including Illusion of Control, Predictive Control, and Interpretive Bias.
State measures	
VAS for Gambling Craving	Single-item measure of momentary gambling desire using a 5-point Likert scale.
Gambling Craving Scale (GACS) (44, 45)	9-item multidimensional assessment of gambling craving on a 5-point scale measuring Anticipation, Desire, and Relief.
VAS for State Anger	Single-item measure of momentary anger using a 5point scale.

Following group assignment, BITalino sensors will be positioned according to standardized protocols (see Appendix D, **Figure 2**): Ag/AgCl electrodes for electrodermal activity positioned on the palm of the non-dominant hand; photoplethysmography sensor placed on the index fingertip of the non-dominant hand to capture volumetric changes in blood. A three-minute resting baseline is recorded under low-stimulation conditions.

Before experimental exposure, participants will complete a brief interactive tutorial to familiarize themselves with the VR environment.

Each participant will begin with 10000 virtual credits (equivalent to €100) and can select among four predefined bet levels: 25, 50, 100, or 200 credits. The VR gambling task will consist of five sessions presented in a predetermined order across participants, each isolating a specific structural slot-machine feature (as described in the Introduction).

Throughout the VR exposure, research staff will continuously monitor for symptoms of cybersickness or distress. Participants may terminate the session at any time using an emergency button (ESC). The session will be immediately discontinued if participants will report intense discomfort, explicitly request to stop, or activate the emergency exit button. Emergency protocols will be established for managing severe reactions, with appropriate referral procedures for participants requiring additional support. The maximum VR exposure duration will be of 20 minutes, in accordance with safety guidelines to reduce adverse effects, particularly visual strain and headaches.

**Figure 6:**
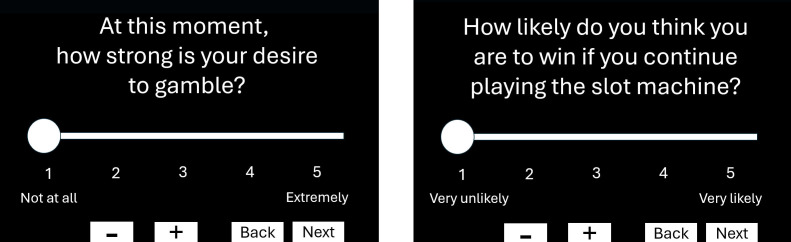
The two VAS questions presented during the Reflection Group sessions: on the left current gambling craving, and on the right perceived probability of winning.

In the Flow Group, participants will complete the five VR sessions consecutively without interruption (approximately 15–20 minutes). In the Reflection Group, brief pauses will occur between sessions during which two VAS items assess on-time craving and perceived winning probability as reported in **Figure 6**. The Break Group will follow the same procedure as the Reflection Group, but a loading screen will appear between sessions instead of the VAS items.

These interruptions intentionally disrupt immersive continuity and are expected to attenuate flow states. The total duration of this condition will therefore be slightly longer, approximately 20 minutes.

Across all sessions, continuous multimodal recording will capture behavioral indices (betting patterns, reaction times, spin frequency, decision latency), physiological measures (skin conductance, photoplethysmography), and eye-tracking metrics (fixation patterns, saccades, pupil diameter). All streams are synchronized with millisecond-precision event markers linked to gambling outcomes (see Appendix C for the full set of measurements).

Immediately after VR exposure in order to evaluate changes from baseline, participants will complete the post-test battery, including all state-dependent measures (State Measures in **Table 5**), the GRCS-RA, and the ITC-Sense of Presence Inventory (VR Experience in **Table 6**). A detailed overview of all tests is reported in **Table 7**.

**Table 6:** ITC–Sense of Presence Inventory (ITC-SOPI) assessing subjective experience of presence in the virtual environment.

Measure	Description
VR Experience	
ITC–Sense of Presence Inventory (ITC-SOPI) (46)	44-item assessment of subjective presence experience across four dimensions: Spatial Presence, Engagement, Ecological Validity, and Negative Effects.

It is included in the post-test assessment.

**Table 7:** Detailed overview of psychological measures administered across study phases.

Study phase	Measure	Construct	Items	Response scale	Subscales/output	Purpose
Online prescreening	Socio-demographic questionnaire Medical history questionnaire	Sociodemographic background Contraindications to VR exposure	– –	Categorical /open-ended Yes/No, checklist	Descriptive variables Eligibility screening	Sample characterization and stratification. Safety screening for VR exposure.
	Canadian Problem Gambling Index	Gambling risk	9	4-point frequency	Total score	Screening for at-risk or problematic gambling.
	SCL-90 Psychoticism sub- Scale	Psychoticism screening	10	5-point intensity	Subscale score	Exclusion of severe psychological vulnerability.
	Ten-Item Personality Inventory–Revised	Personality traits (Big Five)	10	7-point agreement	Five trait scores	Baseline personality profiling.
	16-item BIDR	Social desirability	16	6-point agreement	Two subscale scores	Assessment of socially desirable responding and control for self-presentation bias.
Pre-test (laboratory)	VAS for Gambling Craving Gambling Craving Scale GRCS–Revised for Adolescents	Momentary gambling cravingGambling craving Gambling-related cognitive beliefs	1 9 14	5-point Likert 5-point agreement 5-point agreement	Single item Anticipation, Desire, Relief Illusion of Control, Predictive Control, Interpretive Bias	Baseline assessment of craving. Baseline multidimensional craving assessment. Baseline assessment of relatively stable cognitive beliefs.
	Depression Anxiety Stress Scales–21	Psychological distress	21	4-point frequency	Depression, Anxiety, Stress	Baseline assessment of psychological distress.
	Short UPPS-P Impulsive Behavior Scale	Impulsivity	20	4-point agreement	Five impulsivity dimensions	Baseline assessment of impulsivity-related traits.
	VAS for State Anger	Momentary anger	1	5-point Likert	Single item	Baseline assessment of emotional state.
During VR exposure	VAS for Gambling Craving VAS for Perceived Probability of Winning	Momentary gambling craving Perceived winning probability	1 1	5-point Likert 5-point Likert	Single item Single item	State assessment during task interruptions (Reflection Group only). Cognitive appraisal during task interruptions (Reflection Group only).
Post-test (laboratory)	VAS for Gambling Craving Gambling Craving Scale	Momentary gambling craving Gambling craving	1 9	5-point Likert 5-point agreement	Single item Anticipation, Desire, Relief	Post-exposure assessment of craving. Post-exposure multidimensional craving assessment.
	GRCS–Revised for Adolescents	Gambling-related cognitive beliefs	14	5-point agreement	Illusion of Control, Predictive Control, Interpretive Bias	Post-exposure assessment of cognitive beliefs.
	VAS for State Anger	Momentary anger	1	5-point Likert	Single item	Post-exposure assessment of emotional state.
	ITC–Sense of Presence Inventory	Presence and negative effects	44	Likert-type agreement	Spatial Presence, Engagement, Ecological Validity, Negative Effects	Assessment of subjective VR experience and negative effects.

#### 2.4.3 Debriefing and closing procedure

At the end, all participants will view an explanatory video that provides an overview of the cognitive, emotional, and structural mechanisms involved in slot-machine gambling (corresponding to the seventh block in **Figure 5**). Although the SAFESLOT gambling environment is entirely fictitious, it may elicit heightened physiological arousal; therefore, a structured debriefing session with support of the video will be conducted by a psychologist from the research team to contextualize the cognitive and affective conditioning mechanisms underlying gambling addiction. During this, topics including GD symptoms and warning signs, slot machine functioning, and the role of cognitive distortions are addressed, with the goal of increasing participants’ awareness of risks and consequences associated with problematic gambling. Moreover, participants will undergo guided training in diaphragmatic breathing techniques for emotional self-regulation. To strengthen the overall preventative intervention, participants will also receive an informational booklet containing QR codes that allow them to revisit the psychoeducational materials, access supplementary resources on slot machine mechanisms and emotional-regulation strategies, and consult the website of the regional National Health Drug Services, with contact information for specialized support if needed.

### 2.5 Participants

The sample will consist of approximately 200 university students recruited primarily from bachelor’s degree programs at the University of Florence. All study procedures have received approval from the Ethics Committee of the University of Florence (Protocol Number: 0260915). Participants will provide written informed consent after receiving comprehensive information about study procedures, potential risks (including motion sickness or psychological discomfort), data handling protocols, and their right to withdraw at any time without penalty. The consent process emphasizes that participation is voluntary and that the study involves simulated gambling with virtual currency only, with no real monetary gains or losses.

Participants will be eligible for inclusion if they are between 18 and 65 years of age, able to provide informed consent, and have no contraindications for virtual reality exposure. Exclusion criteria are designed to protect vulnerable populations and ensure data quality, following established protocols for VR research. **Table 8** details the complete exclusion criteria.

**Table 8:** Exclusion criteria for participant eligibility for the immersive SAFESLOT VR gambling simulation.

Specific Criteria	Specific Criteria
Medical Criteria	• Epilepsy or family history of epilepsy (due to potential seizure risk from head-mounted displays); • Migraine or other neurological disorders (like schizophrenia) associated with increased visual discomfort or VR intolerance (8, 40); • Hearing loss that would impair interaction with VR audio components; • Low vision that would impair interaction with VR visual components; • Cardiovascular problems that could affect psychophysiological measurements; • Vestibular disorders that may increase risk of falls or motion sickness during VR exposure (47); • Onset of intense cybersickness during the session that prevents continuation of the VR experience (ongoing exclusion criterion).
Psychological Criteria	• Presence of psychotic symptoms as assessed by the PSY subscale of the SCL-90 (48); • Score of 1 or higher on the CPGI, indicating at-risk or problem gambling behavior.

## 3 Data analysis

To ensure alignment between study aims, hypotheses, and analytical strategy, analyses are organized into confirmatory and exploratory components. Confirmatory analyses are designed to test the primary hypotheses regarding the effects of experimental condition (Flow, Reflection, Break) and outcome type on behavioral, physiological, and self-report measures. Exploratory analyses aim to identify complex multimodal patterns and generate hypotheses for future research.

Behavioral, physiological, and eye-tracking data will be synchronized using millisecond-precision event markers corresponding to gambling outcomes. Skin conductance signals will be filtered and corrected for artifacts, and phasic skin conductance response (SCR) amplitudes will be extracted time-locked to each gambling outcome. Cardiac signals will be processed to derive heart rate (HR, in bpm) and heart rate variability (HRV) indices, with time-domain measures computed over standardized temporal windows during task execution. Eye-tracking data will be preprocessed to remove noise and tracking loss and used to extract gaze direction, fixation duration, saccadic activity, pupil diameter and spontaneous blink rates (both during task execution and rest periods). Physiological variables will be standardized within participants to account for inter-individual variability. Reaction times (RTs) will be defined as the latency between the presentation of a gambling outcome (i.e., end of reel spin) and the initiation of the subsequent action (e.g., next spin or bet adjustment). RT distributions will be inspected for outliers and log-transformed where appropriate.

Classical statistic analytic techniques such as correlations and regressions, independent t-tests and mixed ANOVAs will be applied to analyze respectively the relationships between self-report, behavioral, and physiological methods, and to understand the effect of the virtual slot machine experience on gambling-related cognitive distortions and craving, also considering the manipulation between flow, reflection, and a break experience.

As a preliminary step, associations between physiological activation (SCR amplitude and HRV), behavioral responses (RTs), and self-report measures (craving and gambling-related cognitive beliefs) will be examined using correlational analyses. This step will test whether RTs are robustly and negatively associated with physiological arousal and subjective measures, thereby supporting their use as an integrative behavioral index of gambling-related reactivity. These correlational analyses will also be conducted separately for each experimental condition (Flow, Reflection, and Break) to examine whether the strength of associations between RTs and physiological/self-report indices differs as a function of uninterrupted gambling, gambling interrupted with self-reflective prompts, and gambling interrupted without reflection. Stronger correlations in the Flow Group compared to the Reflection Group would indicate tighter coupling between arousal and behavioral responses during sustained immersion, consistent with enhanced automatic processing. Regarding the Break Group, intermediate correlations (lower than in the Flow Group, but higher than in the Reflection Group) would reflect a partial attenuation of automatic arousal-behavior coupling due to interruptions alone, in absence of self-reflective prompts.

Following confirmation of these associations, trial-level behavioral data (i.e., reaction times recorded for each individual slot-machine spin) will be analyzed using linear mixed-effects models to account for the nested structure of repeated observations within participants. Reaction time following each gambling outcome will serve as the primary dependent variable. Fixed effects will include outcome type (near miss, large win, large loss, small loss, frequent small win) and experimental condition (Flow, Reflection, and Break), as well as their interaction.

The critical test of the experimental manipulation will be the interaction between outcome type and condition, examining whether uninterrupted gambling (Flow Group) is associated with stronger differentiation of RTs across outcome types compared to gambling interrupted with self-reflective prompts (Reflection Group), and gambling interrupted without reflection (Break Group). A reduced modulation of RTs is expected to follow a graded pattern across groups, being strongest in the Flow Group, weakest in the Reflection Group, and intermediate in the Break Group. This pattern would indicate a progressive attenuation of automatic behavioral responding, with interruptions alone producing partial disruption and reflective interruptions producing a more pronounced attenuation.

Pre-post changes in gambling craving and gambling-related cognitive beliefs will be examined using linear mixed-effects models with Time (pre vs. post) and Group (Flow, Reflection, and Break) as fixed effects, including their interaction. Prior to these analyses, baseline equivalence between groups will be assessed with respect to gambling behavior, gambling-related cognitive beliefs, craving, impulsivity, psychological distress, and social desirability. If groups are equivalent at baseline, these variables will be included as covariates to examine their contribution to individual differences in change scores rather than to control for group imbalance.

Individual differences in pre-post changes in craving and gambling-related cognitive beliefs will subsequently be examined as predictors of behavioral reactivity during play, allowing characterization of response profiles associated with short-term increases in gambling-related risk indicators.

According to the study objectives, machine learning approaches will be employed in an exploratory manner to examine patterns in multimodal data. Unsupervised clustering techniques will be used to identify latent subgroups characterized by distinct response profiles, while supervised models will be applied to explore the potential predictive value of physiological and behavioral features. These analyses are intended to complement hypothesis-driven statistical testing.

All analyses will be conducted using R, MATLAB and Python.

## 4 Expected results

The SAFESLOT protocol is expected to reveal systematic relationships between physiological activation, behavioral responses, and gambling-related cognitive and motivational processes during slot-machine play. Consistent with the hypotheses outlined in the Introduction, we expect to find high and significant negative correlations between the indicators of physiological activation and time reactions after near misses, large initial wins, large losses, small losses, frequent small wins. Faster reaction times are expected to reflect heightened automatic behavioral reactivity in response to outcome salience.

At the behavioral level, the experimental manipulation is expected to modulate these associations. Specifically, linear mixed-effects models are expected to reveal a significant interaction between outcome type and experimental condition (Flow, Reflection, and Break), indicating stronger differentiation of reaction times across outcome types under different gambling conditions. Participants in the Flow Group are therefore expected to show more pronounced modulation of reaction times as a function of gambling outcomes, reflecting tighter coupling between outcome-related arousal and automatic behavioral responding during sustained flow states. In contrast, participants exposed to frequent interruptions in the Reflection Group are expected to show attenuated differentiation of reaction times across outcome types, consistent with reduced immersion and increased reflective processing. Participants in the Break Group will likely show intermediate modulation, consistent with a partial disruption of automatic responding due to interruptions alone.

Furthermore, participants who show greater increases in gambling-related craving and cognitive distortions from pre-test to post-test are hypothesized to show heightened physiological activation and faster behavioral responses during gameplay, forming a psychophysiological profile associated with elevated short-term risk. In contrast, participants in the Reflection condition should reveal lower increases in craving and gambling-related cognitive distortions, suggesting a modulatory effect of brief reflective pauses on gambling-related reactivity, beyond the effect of interruptions alone. These effects are expected to emerge after accounting for baseline equivalence between groups with respect to gambling behavior, gambling-related cognitive beliefs, craving, impulsivity, and psychological distress, with baseline measures contributing to individual differences in change trajectories rather than reflecting pre-existing group imbalance.

Overall, the possible results could support the utility of integrating immersive virtual reality with multimodal measurement to identify behavioral and physiological markers associated with short-term changes in gambling-related risk processes, with implications for early identification and preventive intervention strategies.

## 5 Conclusions

The SAFESLOT project presents an integrated approach for investigating gambling-related cognitive distortions, craving, and psychophysiological responses in a controlled and ecologically valid virtual reality environment. By integrating behavioral, physiological, and self-report measures, summarized in **Table 7**, the protocol allows a detailed, multimodal characterization of moment-to-moment gambling processes and their modulation by experimental manipulations such as flow disruption.

This approach addresses important methodological limitations in previous gambling research, including reliance on static self-report instruments, limited ecological validity, and under-explored at-risk populations such as university students. By combining immersive VR with continuous measurement and computational modeling, SAFESLOT has the potential to identify behavioral and physiological markers of short-term changes in gambling-related risk indicators.

Furthermore, the experimental manipulation and multimodal design provide a basis for evaluating potential preventive strategies, such as brief reflective pauses, that may attenuate cognitive distortions and craving during gambling. The protocol also lays the groundwork for future studies employing predictive and machine learning approaches to uncover latent patterns of risk and resilience.

Some limitations should be acknowledged. First, the use of virtual currency may attenuate financial risk perception compared to real-money gambling. Second, the student sample, while relevant for prevention research, may limit generalizability to clinical populations. Third, the cross-sectional design precludes examination of whether acute psychophysiological responses predict long-term gambling outcomes. Fourth, certain design choices adopted to ensure experimental control and feasibility may limit ecological validity. In particular, the use of a fixed 3-second interval between spins, while methodologically useful for isolating variables of interest and ensuring comparability across participants, does not reflect the temporal variability of commercial slot machines, where outcome timing is often manipulated to enhance uncertainty and engagement. Similarly, the proportion of positive outcomes in the present slot machine is higher than that typically observed in commercial systems, which are usually set just below 50% of trials. This choice was intentionally made to ensure sufficient exposure to rewarding events and to elicit measurable psychophysiological responses within the relatively short duration of the experimental session. Finally, we recognize that, although functional to study the effects of gambling experience on human cognition and behavior, the implementation of pre-test measurement session immediately before the SAFE SLOT experience may have some effects in terms of participants’ reflection and self-awareness. Future studies should aim to incorporate greater temporal variability and more realistic reward structures, thereby enhancing ecological validity and more closely approximating real world gambling environments. These limitations suggest directions for future research employing real-stakes paradigms and longitudinal designs.

Overall, SAFESLOT contributes both methodologically and conceptually to the study of gambling behavior, offering a replicable framework for early detection in gambling-related harm. If validated, the psychophysiological markers identified through SAFESLOT could enable real-time risk detection systems in commercial gambling environments, providing objective indicators to complement self-report screening. The protocol’s modular design allows adaptation to other gambling formats (i.e., sports betting, poker etc.) and populations (i.e., clinical samples, problem gamblers in treatment etc.). Most critically, the demonstration that brief reflective pauses attenuate craving and cognitive distortions may offer an evidence-based strategy for responsible gambling interventions implementable at scale through mandatory ‘reality checks’ in online platforms.

## Glossary

ANOVA: Analysis of variance
CPGI: Canadian Problem Gambling Index
DASS-21: Depression Anxiety Stress Scales–21 items
EDA: Electrodermal Activity
EGM: Electronic Gaming Machine
GD: Gambling Disorder
GACS: Gambling Craving Scale
GRCS: Gambling Related Cognitions Scale
GRCS-RA: Gambling Related Cognitions Scale–Revised for Adolescents
HR: Heart Rate
HRV: Heart Rate Variability
LDW: Loss Disguised as Win
PPG: Photoplethysmography
RT: Reaction Time
SCR: Skin Conductance Response
VAS: Visual Analogue Scale
VLT: Video Lottery Terminal
VR: Virtual Reality.
