# Supplementary material for: SAFESLOT: an experimental virtual reality protocol to examine flow, craving, and cognitive biases in gambling
Source: Front Psychiatry. 2026 May 8;17:1787985. doi: 10.3389/fpsyt.2026.1787985 (PMC13194411; doi:10.3389/fpsyt.2026.1787985)
Supplement: Supplementary file 1 [file DataSheet1.pdf]

A Hardware Specifications

The SAFESLOT system integrates advanced hardware components for an immersive VR experience and multimodal data acquisition:

- **Vive Focus Vision Headset**: PCVR/Standalone headset (Fig. 1, left) featuring dual LCD panels (2 448 x 2 448 pixels per eye, up to 120 Hz refresh rate). The device includes four embedded tracking cameras supporting eye-tracking and hand-tracking, as well as a gyroscope, proximity sensor and depth sensor, enabling accurate monitoring of head motion, gaze behavior, and interaction within the virtual environment.
- **BITalino (r)evolution Biosignals Platform**: Research-grade physiological monitoring system (Fig. 1, right) featuring Photoplethysmography (PPG) sensor for blood volume changes, and electrodermal activity (EDA) sensor for skin conductance response. All sensors use pre-calibrated Ag/AgCl pre-gelled disposable electrodes with continuous recording at 100 Hz, synchronized with game events.

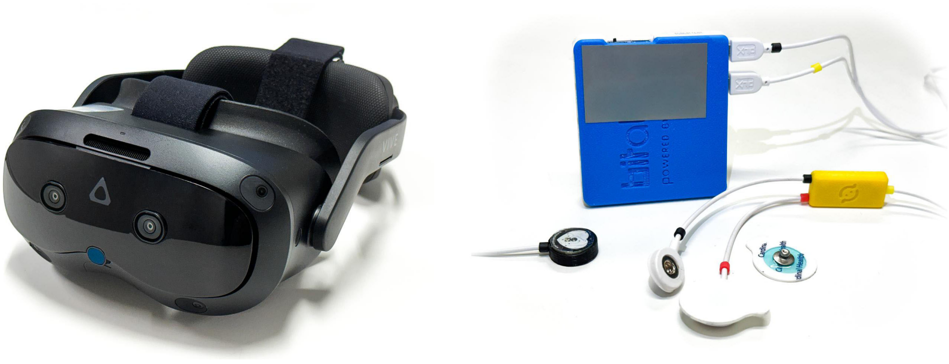

Fig. 1 Vive Focus Vision Headset and BITalino (r)evolution Biosignals Platform with photoplethysmography (PPG) and electrodermal activity (EDA) sensors.

B VR environment and implementation

B.1 Virtual Environment

The VR slot-machine room is developed in Unity 6. The environment recreates a typical Video Lottery Terminal (VLT) room with six slot machines, carpeted flooring, dim lighting, and stools commonly found in land-based venues (see Fig. 2).

The SAFESLOT system will integrate VR hardware Vive Focus Vision Headset with multimodal physiological recording BITalino (r)evolution Biosignals Platform. All physiological signals will be recorded at a sampling rate of 100 Hz.

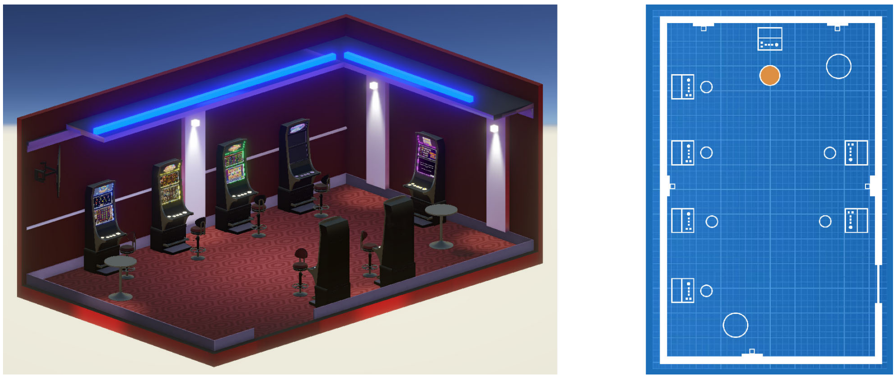

Fig. 2 Isometric view and floor plan of the SAFESLOT slot machine room. The figure illustrates the spatial layout of the VR environment, including the positions of all slot machines. The participant's location during the experiment is highlighted in orange to indicate the point of interaction with the active slot machine.

B.2 Control panel details

SAFESLOT software simulates a standard commercial on-line slot machine with five reels and three visible symbols each. The participant can adjust bets, active paylines, access general info and initiate spin through the control panel in Fig. 3 whose elements' description is in Tab. 1.

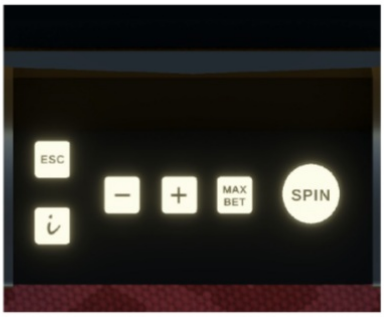

Fig. 3 SAFESLOT control panel.

Table 1 Description of VR controls in the slot machine simulation.

Button	Function
Spin	Primary action button initiating each game round. Participants press this button to start the reel rotation after selecting the wager amount.
Max Bet	Single-press button that automatically sets the wager to the maximum available level (200 credits), replicating a common feature in commercial slot machines designed to promote higher-risk gambling behavior.
Bet Adjustment Controls	Two buttons allowing participants to increase (+) or decrease (-) the wager amount across four predefined bet levels (25, 50, 100, and 200 credits).
Information Button (i)	Opens an informational overlay displaying the paytable, including winning symbol combinations, corresponding payouts relative to the current bet, and active payline configurations.
Emergency Stop Button (Esc)	Allows participants to immediately exit the gambling simulation at any time without providing justification. Activation terminates the VR session and alerts research staff, ensuring participant safety and compliance with ethical guidelines.

C Data Acquired

All user interactions are logged with millisecond precision, including bet changes, button presses, spin initiation, and reaction times following outcomes.

Behavioral Measures

Continuous monitoring during the VR-session of game behaviors provides objective indicators of gambling engagement and decision-making patterns: betting amounts for each spin, inter-spin latency (reaction time between outcome presentation and next bet), total session duration, bet level selections (25, 50, 100, or 200 credits), variability

in betting patterns following wins, losses, and near-misses, and utilization of early stop button to terminate sessions prematurely.

In particular, the following behavioral aspects of each participant are gathered:

- *Reaction time*: The time between consecutive spins is collected as representative of the subject reaction to the spin event. The sum of the subject reaction times is the session duration;
- *Betting amount*: Before each spin, the bet change/persistence is collected. If the subject changes his bet several times, only the last one is recorded;
- *Spin outcome*: The final outcome is recorded, both in terms of credits loss or gain and in terms of the symbols' sequence.

Physiological Measures

Physiological signals are acquired using the BITalino (r)evolution platform. Electrodermal activity (EDA) and photoplethysmography (PPG) signals are recorded continuously and synchronized with in-game events.

Measures include:

- *Electrodermal Activity (EDA)*: Skin conductance is measured using two pre-calibrated Ag/AgCl electrodes positioned on the palm of the non-dominant hand following standard placement protocols (Fig. 4). The measurement range is $0-25\mu S$ and signal acquisition occurs at a 100 Hz sampling frequency;
- *Photoplethysmography (PPG)*: The system captures volumetric changes in blood at a sampling frequency of 100 Hz using a fingertip sensor with a green light (wavelength of $\sim$ 520 nm) placed on the index finger of the non-dominant hand (Fig. 4). During the data analysis phase, heart rate (HR, in bpm) and heart rate variability (HRV) are derived from this signal.

Eye-Tracking Metrics

The integrated binocular eye-tracking system in the Vive Focus Vision (60 Hz sampling rate) enables continuous monitoring of oculomotor responses throughout gameplay:

- *Fixation Patterns and Gaze Distribution:* Spatial mapping of gaze locations across the virtual environment, quantifying attention directed toward gambling-relevant versus peripheral stimuli. Fixation positions are captured in Unity as three-dimensional coordinates (x, y, z) expressed in meters (m);
- *Pupil Diameter:* Pupillometry measures changes in pupil size as indicators of cognitive load, arousal, and emotional responses to gambling outcomes. Pupil dilation has been associated with increased cognitive processing demands and autonomic activation. During the data analysis phase, changes in pupil diameter in response to stimuli will be extracted from the continuous pupil size recordings. Measurements are expressed in millimeters (mm);
- *Blink Rate and Duration:* Spontaneous blinking patterns may reflect cognitive effort, stress levels, and immersion in the virtual environment. Eye openness ranges from 0 (fully closed) to 1 (fully open).

All eye-tracking data are preprocessed to remove artifacts, normalize for individual baseline differences, and extract event-related metrics time-locked to specific gambling outcomes (wins, losses, near-misses, LDWs).

D Laboratory Session Timeline

Upon entering the laboratory, participants are required to set to a computer terminal and receive a detailed explanation of the study procedures.

Phase 1: Inclusion Criteria (20 minutes)

Participants must provide the written informed consent for the laboratory VR-session. Research team review potential risks, including motion sickness and psychological discomfort, and emphasize the participants' right to withdraw at any time without penalty. Following consent, participants complete the pre-test questionnaire detailed in the Experimental Phase of the Methods section.

Phase 2: Experimental Phase (20 – 30 minutes)

At the beginning of this phase, eligible participants are assigned to one of three experimental conditions (Flow, Reflection or Break) using computer-generated randomization to ensure balanced group allocation. Following group assignment, BITalino sensors are positioned according to standardized protocols, as shown in Fig. 4

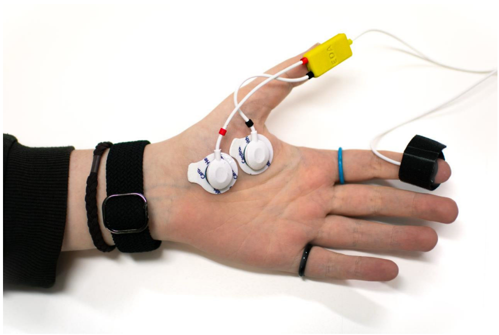

Fig. 4 Placement of EDA Electrodes and PPG sensor on the non-dominant hand.

Participants wear the headset with adjustment for optimal comfort and visual clarity (IPD calibration 57 - 72 mm). Five-point eye-tracking calibration ensures measurement accuracy. Before tutorial session, participants read standardized explaining the slot machine operational principles. These instructions cover:

- the credit system and virtual currency (10 000 credits equivalent to €100);
- bet level options and adjustment controls;
- winning mechanics, including symbol combinations required for payouts;

- payline configurations and how winning combinations are evaluated across the five fixed paylines;
- the paytable structure showing payout multipliers for different symbol combinations;
- the role of wild symbols in completing winning combinations;
- interface navigation and control panel functions.

This preliminary briefing ensures that all participants begin with the same understanding of game mechanics, minimizing confusion and facilitating direct comparison of gambling behaviors across participants.

The first session serves as an interactive tutorial, allowing participants to familiarize themselves with the virtual environment through hands-on practice. During this phase, participants explore spatial navigation within the virtual environment, test hand-tracking responsiveness and button functions, and execute several trial spins to experience the complete gameplay cycle (bet selection, spin button, outcome presentation, credit updates). The tutorial includes guided prompts explaining key interface elements as they are encountered, reinforcing the operational rules of the slot machine through direct experience. This training phase minimizes disorientation, reduces cognitive load during subsequent experimental sessions, and ensures adequate comprehension of gameplay mechanics before data collection begins.

Participants complete five slot machine sessions consisting of 30 spins each in the immersive virtual environment. Each participant starts with 10 000 virtual credits and can select from four bet levels after each spin. At the end of the task, the experimental procedure terminates automatically, and the conclusion of the session is clearly indicated to the participant through an on-screen message. Following task completion, dedicated research staff assist participants in safely removing the VR headset and physiological sensors, ensuring comfort and maintaining data collection integrity.

Immediately after the VR-session, participants complete the post-test questionary as described in the Post-test section.

Phase 3: Debriefing and Closing Procedure (15 minutes)

Each participant receives a *University of Florence* gadget as acknowledgment for their time and commitment, with explicit communication that this recognition is independent of virtual gambling outcomes. Participants receive also an informational booklet with QR codes providing access to psychoeducational content on gambling disorder, resources on gambling mechanisms and emotional regulation, and contact details for regional addiction services (SerD). Participants are thanked for their contribution to the research and offered the opportunity to ask any final questions before leaving the laboratory.

E Safety and Hygiene Measures

Given the shared use of VR equipment and physiological monitoring sensors across multiple participants, comprehensive sanification procedures will be implemented to ensure participant safety (in accordance with the guidelines of the *National Health*

Institute and university health and safety regulations). Between each experimental session, the headset will undergo thorough cleaning following manufacturer recommendations and established VR research protocols. All BITalino physiological monitoring sensors utilize disposable, single-use Ag/AgCl pre-gelled electrodes that will be discarded after each participant session. The reusable sensor hardware components will be cleaned with appropriate medical-grade disinfectant solutions between sessions. Electrode placement areas on participants' skin will be prepared using standard procedures, including cleaning when necessary to ensure optimal signal quality and hygiene.
